# Damage tolerance of six dental zirconias with different translucencies

**DOI:** 10.1080/26415275.2020.1809420

**Published:** 2020-08-25

**Authors:** Cathrine Å. Karlsen, Christian Schriwer, Marit Øilo

**Affiliations:** Department of Clinical Dentistry, Faculty of Medicine, University of Bergen, Bergen, Norway

**Keywords:** Dental ceramics, dental crown, yttria

## Abstract

**Purpose:**

High-translucent dental zirconia has been introduced as a suitable material for anterior monolithic restorations. The material composition differs from traditional 3Y-TZP both with regard to yttria content and grain size. Little is known regarding how these alterations affect other properties than translucency and flexural strength. The aim of this study was to evaluate the crack propagation resistance and hardness of dental zirconias with different yttria content and different manufacturing methods.

**Materials and methods:**

Measurement of hardness (HV2/5) and crack propagation from the indents (damage tolerance) was performed using a hardness tester(Vicker) on a flat polished surface of five crowns from six different commercial dental zirconias; one hard-machined 3Y-TZP, three soft-machined 3-5% yttria-stabilized zirconias and two soft-machined zirconias with ≥5% yttria content.

**Results:**

Damage control varied greatly among dental zirconias with different compositions and fabrication methods. The hard-machined 3Y-TZP had better crack propagation resistance than soft-machined, 3-5% yttria-stabilized zirconias

**Conslusion:**

The ultra-translucent zirconias with ≥5% yttria content had the lowest crack propagation resistance. Hardness is not a suitable indicator for damage tolerance.

## Introduction

**Figure 1.**
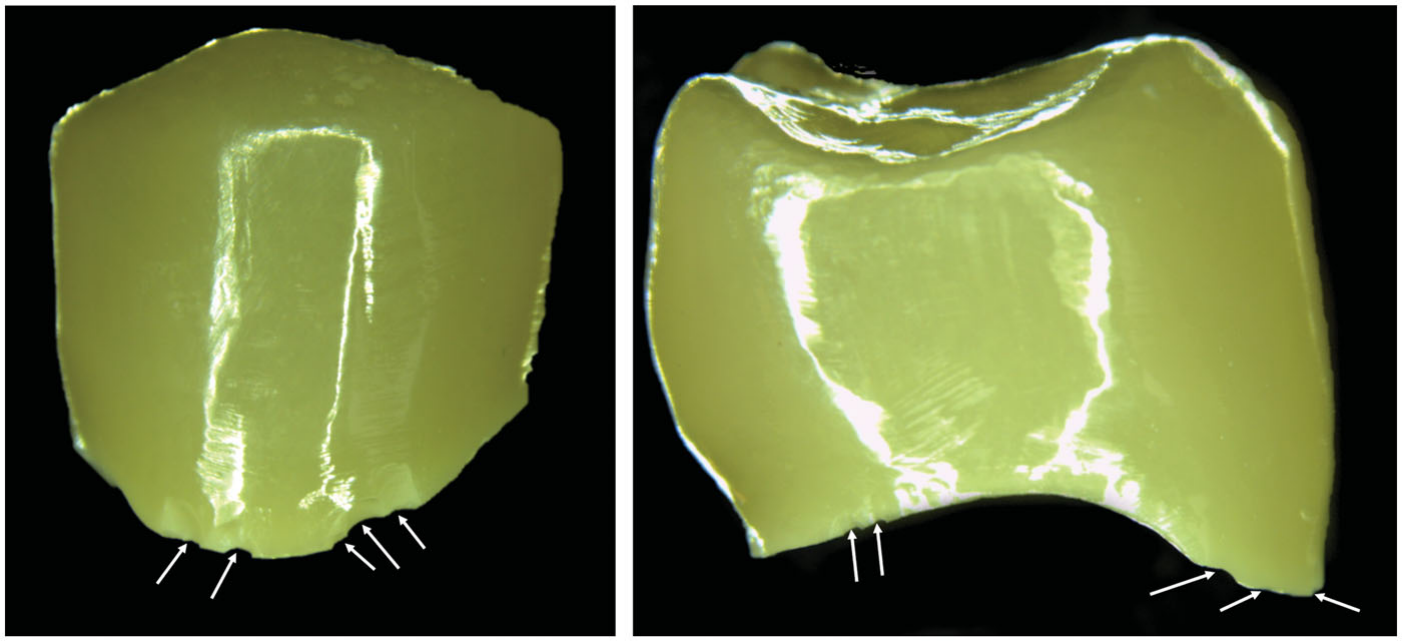
Two examples of ultra-translucent monolithic zirconia crowns delivered from a dental technician with multiple margin flaws (white arrows). These flaws were considered to be due to poor packaging during shipment.

There has been a rapid devolvement in dental zirconia materials towards more translucent and tooth-colored materials [1,2]. The benefit of increased translucency is improved aesthetics, thereby reducing the need for a veneering layer [3–5]. The bi-layered core-veneer structure has shown to be prone to chipping requiring replacement or repair [6–8]. Several commercially available materials are called either translucent, high-translucent or ultra-translucent. The ultra-translucent zirconia usually contains a higher amount of cubic (c) crystals than the less translucent zirconia which consists of predominantly tetragonal (t) crystals [1]. Increasing the amount of stabilizing oxides, such as yttria (YO_2_) leads to a higher content of cubic crystals in the zirconia. Additionally, the sintering temperatures or hold times affect the grain size as well as the crystal lattices [1,9,10]. This will reduce the number of borders where the light transmission can be affected in the passage through the material and thus reduce scattering which makes the material appear white and opaque. These alterations combined with a reduction of the content of alumina (Al_2_O_3_) affect light transmission through the material, but also the fracture strength [1,2,4,11]. There is limited documentation on whether other mechanical properties are affected or not [12]. Personal experience and communication with dentists and dental technicians indicate that margin chipping is more frequent with the ultra-translucent materials (Figure 1). Margin damages can be detrimental for crown strength [13–16].

The aim of this study was to assess the damage tolerance of commercially available dental zirconias with different yttria content. The null-hypothesis was that there was no difference in damage tolerance among different types of dental zirconias.

## Materials and methods

Six dental zirconias with different translucency, yttria content and fabrication method were tested (Table 1). Two batches of one material (ZX) were used to test variance between batches. From each material, five monolithic crowns were produced according to manufacturers’ instructions. The crowns were made to fit an upper jaw premolar prepared with a shallow circumferential chamfer. Crowns were chosen instead of discs or bars in order to have specimens that were produced in five-axis dental milling units and with clinically relevant wall thickness.

**Table 1.** Overview over the materials used in the study with abbreviations, brand names, fabrication method, yttria content and name of manufacturer.

Abbr.	Brand name	Translucency^a^	Fabrication method	Yttria content^a^	Manufacturer
DZ	Denzir HIP	Semi-translucent	Hard-machined	3 mol%	Denzir AB, Skellefteå, Sweden
CX	DD CubeX^2^	Ultra-translucent	Soft-machined	∼5 mol%	Dental Direkt GmbH, Spenge, Germany
ZX	DD Bio ZX^2^	High-translucent	Soft-machined	<3-5> mol%	
BZ	BruxZir Solid Zirconia	High-translucent	Soft-machined	<3-5> mol%	Glidewell laboratories,Newport beach, CA, USA
PS	Prettau Zirconia	High-translucent	Soft-machined	<3-5> mol%	ZirkonZahn, Gais, Italy
PA	Prettau Anterior	Ultra-translucent	Soft-machined	>5 mol%	

^a^Manufacturers information.

**Figure 2.**
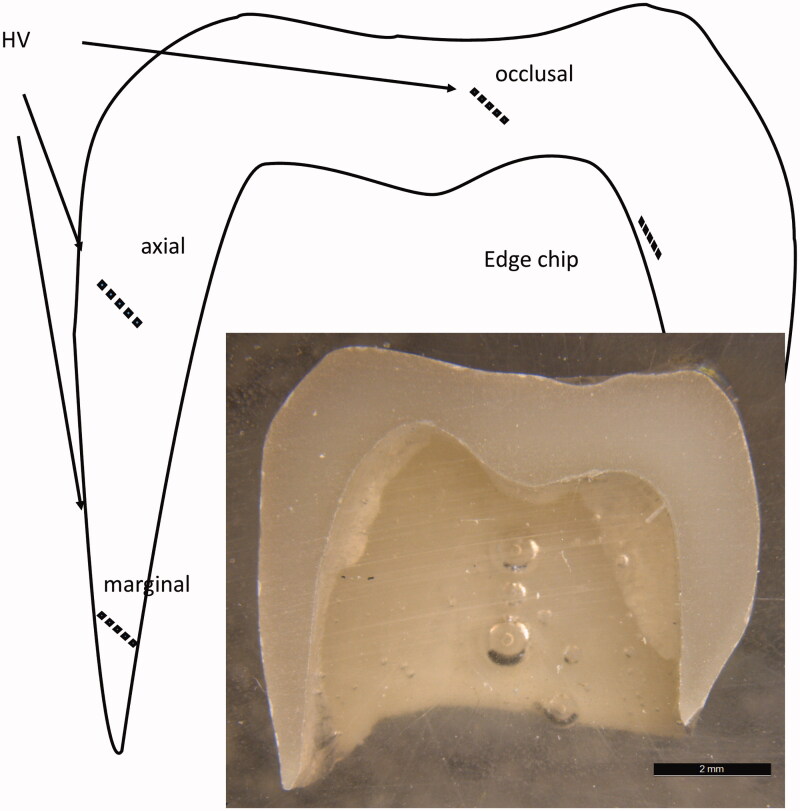
Premolar monolithic crowns embedded in epoxy and cut in half were used for the study. Three regions (marginal, axial and occlusal) were analyzed in each crown.

The crowns were mounted in epoxy molds (Epofix, Struers, Ballerup, Denmark) and the specimens were cut to a flat surface with a diamond cutter of 220 grit as a cross-section of the crown (Figure 2). The surfaces were polished to a high gloss finish in an automatic polishing unit (Tegrapol-11, Struers, Ballerup, Denmark). The polishing sequence started with one minute with a diamond disk of 1200 grit with 10 N pressure and 330 rpm, followed by 2 min with 10 N pressure and 150 rpm with 5 µm diamond paste and 2 min final polishing (3 µm) at 150 rpm. After each step, the crowns were thoroughly rinsed in an ultrasonic bath with soap (Deconex 2%) for ten minutes and steamed clean.

**Figure 3.**
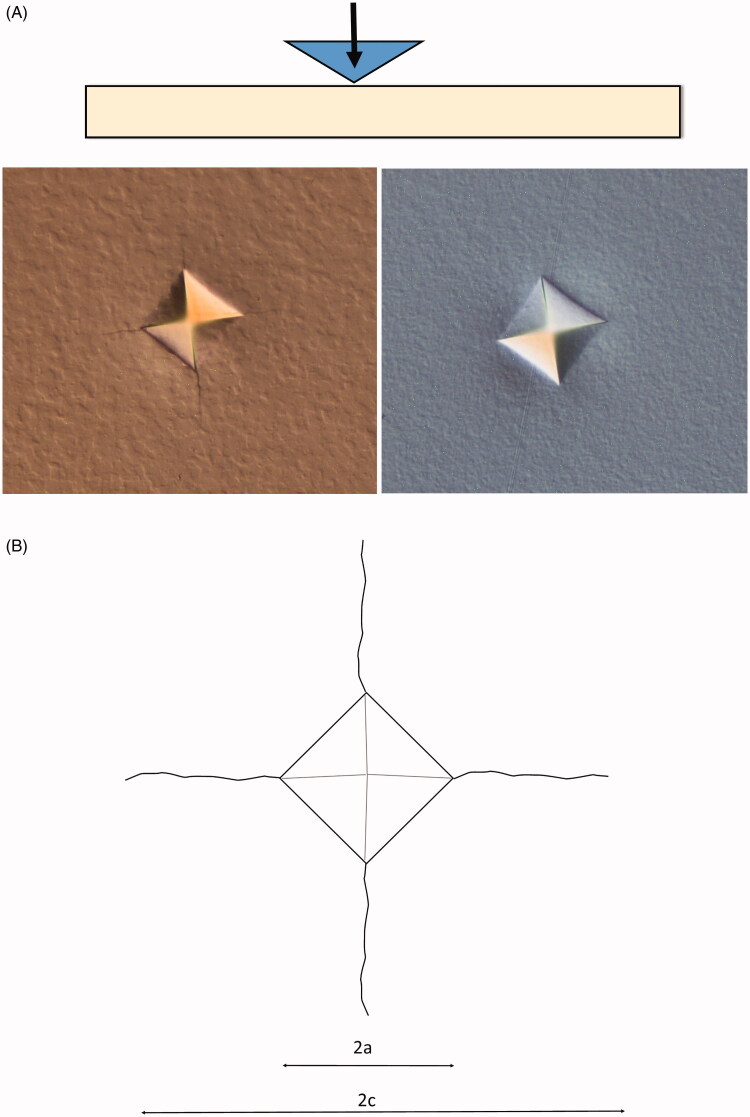
Illustration of the hardness test (A) and the measurement performed (B). 2a is the diagonal length of the indent and 2c is total crack length.

On each crown hardness (Vicker) and crack propagation (damage tolerance) and edge chip tests were performed using a hardness tester (ZHVµ, Zwick Roell, West Midlands, UK). Three separate series of five indentations with two kilograms load for five seconds (HV2/5) with a 136-degree diamond indenter was performed on each crown (Figure 3(A)), five in the marginal region with wall thickness < 0.5 mm, five in the axial wall region with wall thickness between 0.5 and 1 mm and five in the occlusal wall region with wall thickness over 1 mm. Both the diagonal length of the indent and the crack propagating from the indent corners were measured and used in the analyses (Figure 3(B)). The ratio between the crack length and the diagonal length of the indent (*c/a*-ratio) was used as an indicator of damage tolerance. For each location, the mean of the five measurements was registered giving 15 separate values for each test group which was used in the analyses. A series of indents were made gradually closer and closer to the half-cut crowns’ inner walls until a chip was made as an experimental edge chip method [17]. The distance between the 90-degree angle of the test surface and inner wall of the first indent to create an edge chip was measured.

Control measurements of all test methods were performed after one month on 25 randomly chosen spots for each procedure. The same operator performed the controls, but without access to the previous results. The intraclass correlation coefficient (ICC) was >0.9 for indent and crack measurements, indicating a high degree of intra-operator repeatability for these test methods. The mean discrepancy for the repeated measurement was 0.01% (range 0–0.7) for hardness and 0.46% (range 0–10) for crack length measurements. The repeatability of the edge chip method was unacceptably low. The edge chip tests can thus only be used for visual comparison and as a pilot for future studies.

A statistical software package (IBM, SPSS Statistics 25, Chicago, IL, US) was used for analyzing the data. After testing for normality, One-way ANOVA was used for overall-comparison and Tukey’s *post-hoc* analyses for pairwise comparison between groups, with Bonferroni adjustments. The level of significance was set to .05.

## Results

**Figure 4.**
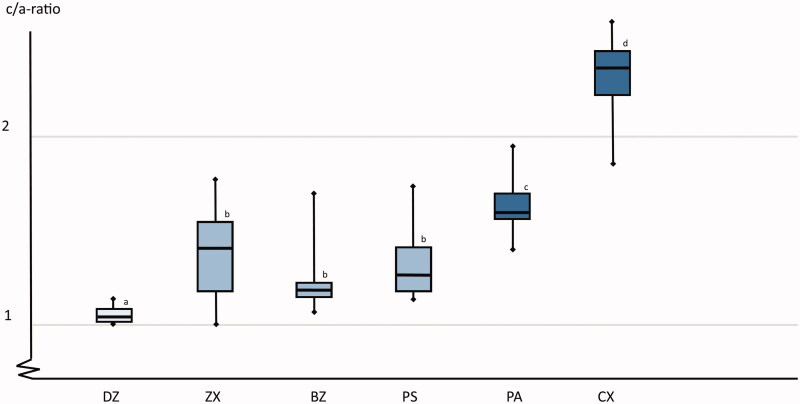
A box plot of the crack/indent-ratio (*c/a*) for the different test groups. Boxes marked with identical superscript letters and not statistically significant different form each other. Horizontal lines represent median values, the boxes represent the interquartile range and the whiskers indicate maximum and minimum values. Boxes marked with same letter are not statistically significant different form each other.

The PA material with the highest content of yttria was statistically significant harder (HV mean 1378, SD 56) than the remaining materials which had very similar hardness (HV mean (SD): DZ 1231 (120), CX 1273 (52), ZX 1269 (102), BZ 1231 (127) and PS 1269 (56)) (*p* < .001). There were statistically significant differences in the c/a-ratio among the materials (Figure 4, *p* < .001). The hard-machined 3Y-TZP material (DZ) had the best crack propagation resistance and the two soft machined materials with ≥5mol% yttria content (PA and CX) had the lowest crack propagation resistance. There were no differences between the two batches of the ZX material, these groups are therefore presented as one group in the following presentations. There were no differences in hardness or *c/a* ratio in the different locations of the crowns, cervical, axial or occlusal.

**Figure 5.**
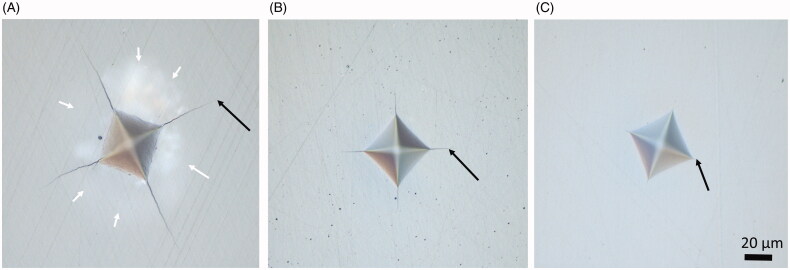
Examples of the variation of the damage zone around the indents as seen in the light microscope. (A) High Yttria content, (B) moderate yttria content, and (C) low yttria content. The arrows indicate end of crack.

**Figure 6.**
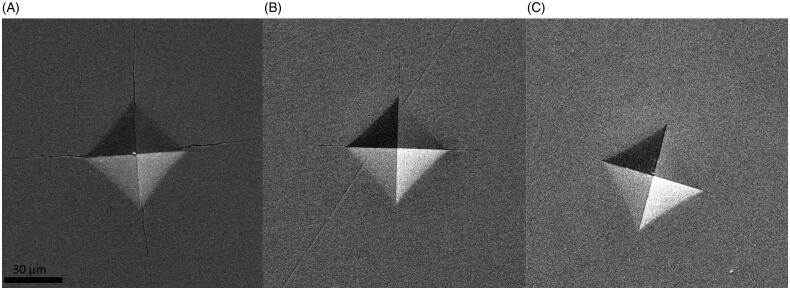
SEM images of typical indentations and cracks in the three different material groups. (A) High yttria content, c/a ratio 2, (B) moderate yttria content, and (C) low yttria content.

**Figure 7.**
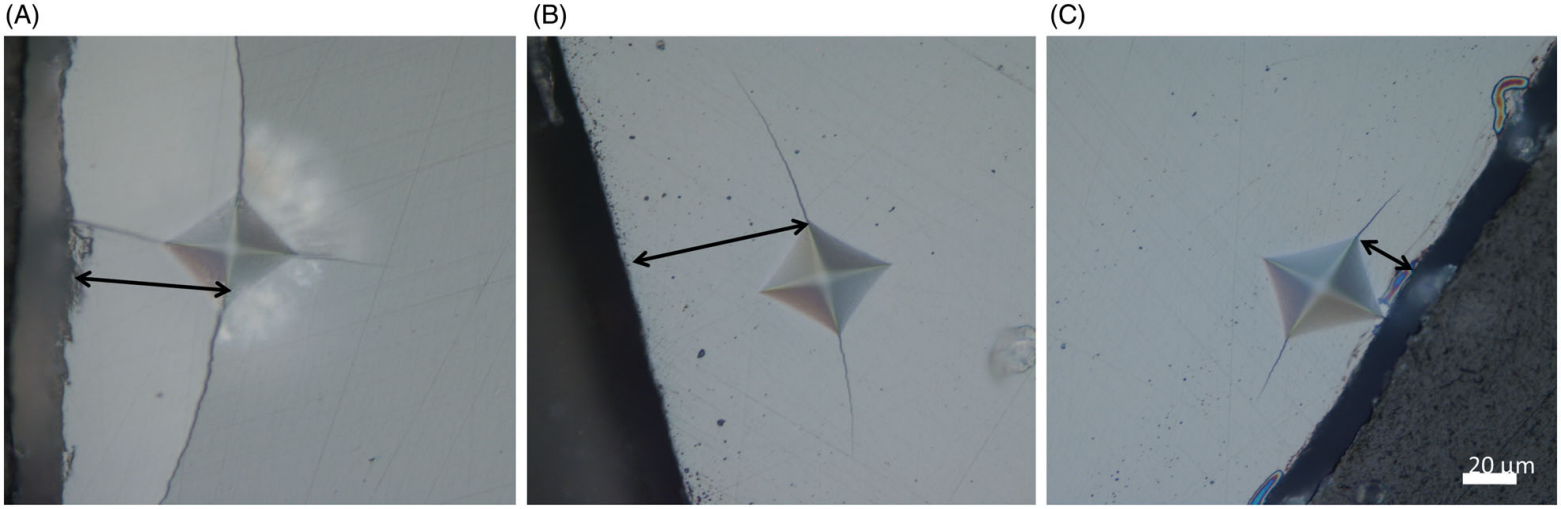
There was apparent differences in how close to the edge an indent could be placed before causing a chip. (A) High yttria content, (B) moderate yttria content, and (C) low yttria content. Arrows indicate distance from the edge.

The damage zone around the indents varied greatly, from no visible damage in the hard-machined zirconia (DZ) to large areas with crushed surface in the two high yttria content (PA and CX) materials (Figures 5 and 6). There were apparent differences among materials in the distance required to withstand an indentation without fracture, but it was difficult to measure the distance to the edge with sufficient precision due to large crushing damages (Figure 7).

## Discussion

The results indicate that although the hardness of dental zirconias with different composition and fabrication methods are quite similar, the damage tolerance varies greatly. Increase in yttria-content reduces the damage tolerance. The hard-machined 3Y-TZP had the best crack inhibiting ability of all the tested materials. Thus, the null-hypothesis is rejected.

The low damage tolerance of the ultra-translucent zirconias (≥5mol% yttria) indicates that these materials are more susceptible to machining damage or transportation damage than the 3-5mol% Y-TZP [14,18–20]. This is in accordance with the clinical experience as shown in Figure 1 and previous studies [1,12,21–23]. How this eventually will affect clinical success is uncertain. It is reasonable to assume that poor damage tolerance reduces fracture resistance and clinical survival time. Furthermore, margin chippings will create uneven margins with increased risk of plaque retention and thus the risk of secondary caries and gingivitis [24]. This should be taken into consideration when setting the machining protocols for the different materials.

Indentation crack provocation tests can be considered as clinically relevant for both the production method and clinical use. High impact from diamonds or metal burs during machining can cause localized stress regions, especially with coarse or worn burs [20,25–27]. Clinically, high-impact damages can be caused by accidental chewing on hard objects in food, such as stone or sand particles in bread. Indentation provocation tests have been used for assessing fracture strength, but the reliability of this method is uncertain as the method does not take into consideration subsurface damage accumulation during indentation [28–31]. The *c/a* ratio seems to be appropriate as a measurement of damage tolerance for zirconia [28,32–34]. This could be a valuable supplement to fracture strength tests performed on discs or bars where the cracks are induced by tension in order to understand the root cause of early failures in the clinic.

Hardness is often reported as a value for comparison among different dental materials. However, the present results clearly shows that hardness is not a suitable variable for comparing strength and fracture resistance of dental zirconia. Increased hardness is not necessarily beneficial as this may increase antagonist wear [35]. This is probably due to the differences in crystal structure among the tested materials. Zirconia appears in three different crystal lattice structures; monoclinic (*m*), tetragonal (*t*) and cubic (*c*). The *m*-structure is unstable and therefore not desirable in dental zirconias. Additions of 3–4 mol% yttria stabilizes the zirconia in a metastable *t*-structure up to a point. During high stress, some crystals may transform back into monoclinic phase. This *t–m transformation* causes localized stress due to a small volumetric difference between *t* and *m* crystal lattices. This *t–m transformatio*n is generally considered as one of the reasons for 3Y-TZP’s good damage tolerance abilities. The explanation for the poorer damage tolerance observed in the present study may be that the cubic crystal structure does not undergo this transformation during stress [36]. The materials with higher yttria content have a higher degree of crystals in the c-lattice structure than 3Y-TZP [1,37]. The present result reveals that although the two ultra-translucent zirconias have the lowest damage tolerance of all the tested materials, there is a difference between these two materials that cannot be explained by material composition alone. There may be other differences in composition or manufacturing processing that are not fully elucidated in the present study, such as differences in grain size and the lattice structure of the tetragonal crystals [10,38].

Given the clinical reports of margin chips, it could be suspected that the zirconia was more brittle in the thin marginal areas than in the thicker occlusal walls. It could be affected by differences in heating and cooling rates. The present finding that no differences were found in the separate locations with different wall thickness indicates that the thickness of the crown wall does not affect neither hardness nor crack resistance. The margin chips are therefore more likely a result of too thin crown margins or machining defects [14]. Unfortunately, we could not reproduce margin chips with the present test method. This needs to be assessed further with a proper standardized edge chip test of appropriate specimens [39]. The challenge of studying dental zirconia is that the machining process is specialized for dental restorations. When producing disks or bars, a different process and equipment is used. It is not evident to what extent the processing affects the mechanical properties of the final product. This is the reason for choosing crown-shaped specimens in the present study.

The present study has some limitations as it only addresses short-term effect of one aspect of fracture initiation of dental zirconia. Aging or dynamic loading has not been taken into consideration either. The number of specimens is limited, but given the low standard deviation and the large differences in results the likelihood of making a type II error of clinical significance is low. The results cannot fully explain the cause of the early failures seen in Figure 1 and further investigations are necessary.

## Conclusion

Increased yttria content results in reduced damage tolerance for dental zirconia Care should be taken in order not to create margin damages during machining, shipping and adjustment. Further studies are necessary to assess whether this will affect clinical survival or not and whether the processing method should be adjusted or not.
